# Ambient Documentation Systems in Emergency Medicine: A Scoping Review of Clinical Precision, Patient Experience, Throughput, and Quality

**DOI:** 10.7759/cureus.106643

**Published:** 2026-04-08

**Authors:** Judith Wolfe, Samantha M Welsh

**Affiliations:** 1 Emergency Medicine, University Hospitals Cleveland Medical Center, Cleveland, USA; 2 Emergency Medicine, Case Western Reserve University School of Medicine, Cleveland, USA

**Keywords:** ambient listening, artificial intelligence, documentation efficiency, documentation quality, ehr management, electronic health record, emergency medicine, multicultural emergency medicine, racial bias, safety and efficacy

## Abstract

This scoping review maps the existing evidence on ambient documentation systems, with an emphasis on emergency medicine applications. It identifies key concepts and knowledge gaps while examining implications for documentation precision, patient experience, clinician well-being, throughput and efficiency, algorithmic equity, safety and governance, and financial and quality outcomes. The review followed the Preferred Reporting Items for Systematic Reviews and Meta-Analyses Extension for Scoping Reviews (PRISMA-ScR) guidelines. The protocol was prospectively registered on the Open Science Framework (OSF). PubMed, MEDLINE (via Ovid), Cumulative Index to Nursing and Allied Health Literature (CINAHL), and Association for Computing Machinery (ACM) Digital Library were searched from January 2015 through March 2026, with final searches conducted on 21 March 2026. English-language peer-reviewed studies, systematic reviews, policy analyses, and expert commentaries addressing ambient documentation systems, medical scribes, documentation burden, automatic speech recognition (ASR) bias, or clinical recording consent in healthcare settings were included. In total, 27 sources met the inclusion criteria. Ambulatory evidence, including two randomized controlled trials and a multicenter pre-post study across six health systems, showed reductions in documentation time, cognitive load, and clinician burnout. Growing emergency department (ED)-specific evidence, with three studies examining ambient artificial intelligence (AI) adoption, documentation time, and note quality in ED settings, suggests that ambient systems may shift rather than eliminate documentation effort, particularly in emergency settings characterized by interruptions, multitasking, and evolving clinical narratives. Computer science literature has identified significant racial and dialect-based disparities in the ASR systems that underpin ambient documentation, with higher word error rates for Black speakers than for White speakers. Key thematic areas include scalability advantages over human scribes, documentation precision, patient consent in high-acuity settings, algorithmic equity, and research gaps in ED-specific outcomes. Ambient documentation systems offer a scalable approach to addressing documentation burden in emergency medicine. The ambulatory evidence base shows consistent benefits in reducing electronic health record time, cognitive load, and clinician burnout, while ED-specific studies support the feasibility of ambient AI, with adoption concentrated in lower-acuity, non-interpreted encounters and ongoing questions about performance in high-acuity, complex settings. Based on these identified themes, we propose a seven-domain framework for evaluating ambient documentation systems in emergency medicine, encompassing documentation precision, patient experience, clinician well-being, throughput and efficiency, algorithmic equity, safety and governance, and financial and quality outcomes. Equitable deployment requires attention to disparities in ASR across racial, ethnic, and linguistic groups, with ongoing monitoring and bias auditing as core governance components. Future research should focus on ED-specific outcomes, including documentation accuracy and patient safety, long-term safety monitoring, patient-centered consent processes, financial and quality implications, and strategies to ensure that ambient documentation benefits all patient and clinician populations equitably.

## Introduction and background

Documentation burden has emerged as a central challenge in modern emergency medicine. Large time-and-motion studies and electronic health record (EHR) log analyses demonstrate that physicians spend a substantial portion of their workday interacting with the EHR rather than in direct patient care. Tai-Seale et al. analyzed over 31 million EHR transactions and found that primary care physicians logged an average of 3.08 hours on office visits and 3.17 hours on desktop medicine each day, with desktop medicine increasing over time while face-to-face patient time declined [1]. Sinsky et al. reported that physicians spent 49.2% of their time on EHR and desk work compared to only 27.0% on direct clinical face time, concluding that for every hour of direct patient care, nearly two additional hours are spent on EHR and desk work, with another one to two hours of personal time each night devoted to computer tasks [2]. In emergency departments (EDs) specifically, Hill et al. found that physicians spent 43% of their time on data entry compared with only 28% on direct patient contact, with total mouse clicks approaching 4,000 during a busy 10-hour shift [3]. Collectively, these studies establish that documentation consumes a significant part of the clinical workday, sometimes exceeding time spent in direct patient care [1-3].

This clerical burden has been strongly associated with clinician burnout, emotional exhaustion, and reduced professional satisfaction [4]. A systematic review and meta-analysis by Wu et al. confirmed a consistent relationship between EHR workload, documentation time, and burnout prevalence across ambulatory healthcare settings, establishing documentation burden as a systemic rather than individual challenge [5]. Importantly, these effects are not static: longitudinal data show that EHR workload has continued to intensify even after the pandemic, with inbox management and "pajama time" documentation demands rising sharply year over year [6]. In EDs, these effects are magnified by frequent interruptions, parallel task demands, and the need to document evolving clinical assessments under time pressure. Arndt et al. found that total primary care physician EHR time per eight hours of scheduled clinic appointments increased by 7.8% from pre-pandemic levels to 2022-2023, with inbox management time rising by 24.4% and after-hours EHR work on unscheduled days increasing by 19.9% [6]. Multicenter studies now confirm that documentation burden remains the primary target for artificial intelligence (AI)-assisted interventions, with ambient AI scribes demonstrating measurable reductions in burnout across multiple health systems [7].

Ambient documentation systems (Abridge, Dragon Ambient eXperience (DAX) Copilot, Nabla) represent a scalable alternative to traditional documentation support models such as human scribes. Unlike human scribes, ambient systems passively capture natural clinician-patient conversation and autonomously generate draft clinical notes for clinician review, without producing diagnoses or treatment recommendations. In addition, where human scribes require recruitment, training, and retention and can serve only one provider at a time, ambient systems can be deployed across an entire health system simultaneously, offering the potential for rapid, organization-wide impact on documentation burden and clinician well-being. The rapid proliferation of these tools has made ambient AI scribes among the most widely deployed generative AI applications in healthcare, with emerging evidence of measurable improvements in physician financial productivity [8].

However, ambient documentation relies on automatic speech recognition (ASR), software that converts spoken words into written text. Recent computer science studies show significant performance disparities across racial, ethnic, and linguistic groups, with word error rates (WERs) that quantify transcription inaccuracy varying by speaker demographics [9]. These disparities raise important questions about whether ambient systems may inadvertently introduce or worsen inequities in documentation quality, particularly in EDs that serve among the most diverse patient populations in healthcare.

This scoping review maps the existing evidence on ambient documentation systems, with an emphasis on emergency medicine, identifies key concepts and knowledge gaps, and examines implications for documentation precision, patient experience, clinician well-being, throughput and efficiency, algorithmic equity, safety and governance, and financial and quality outcomes.

## Review

Methods

Protocol and Registration

This scoping review was conducted and reported in accordance with the Preferred Reporting Items for Systematic Reviews and Meta-Analyses (PRISMA) 2020 statement and the PRISMA Extension for Scoping Reviews (PRISMA-ScR) guidelines [10,11]. The review protocol was prospectively registered on the Open Science Framework (OSF) on January 20, 2026 (https://osf.io/nfqu6) prior to commencing data extraction.

Information Sources and Search Strategy

Four electronic databases were searched: PubMed, MEDLINE (via Ovid), Cumulative Index to Nursing and Allied Health Literature (CINAHL), and Association for Computing Machinery (ACM) Digital Library. The study period spanned January 2015 through March 2026; final searches were executed on 21 March 2026. The 2015 start date was selected to capture the emergence of commercially viable ASR-based documentation tools alongside the intensification of EHR burden literature following widespread EHR adoption.

Search terms were combined using Boolean operators (AND, OR): ("ambient AI" OR "ambient documentation" OR "digital scribe" OR "AI scribe" OR "clinical documentation") AND ("emergency department" OR "physician burnout" OR "electronic health record efficiency" OR "documentation burden" OR "speech recognition bias" OR "speech recognition disparities"). Database-specific controlled vocabulary (e.g., Medical Subject Headings (MeSH) terms in PubMed/MEDLINE) was applied where available. The reference lists of all included studies were hand-searched to identify additional sources not captured by database searches. The full search strategies for each database, including all Boolean combinations and controlled vocabulary terms, are provided in Appendix A.

Eligibility Criteria

Sources were included if they: (1) addressed ambient documentation systems, AI-assisted clinical documentation, medical scribes, documentation burden, disparities in ASR performance, or clinical recording consent in healthcare settings; (2) were peer-reviewed original research (randomized trials, cohort studies, cross-sectional studies), systematic reviews, policy analyses, or expert commentaries; (3) were published in English; and (4) were published between January 2015 and March 2026.

Sources were excluded if they: (1) focused exclusively on nonclinical settings; (2) did not address clinical documentation technology or speech recognition equity; (3) were conference abstracts without full-text availability; or (4) were non-peer-reviewed organizational reports or grey literature.

Selection of Sources of Evidence

**Figure 1:**
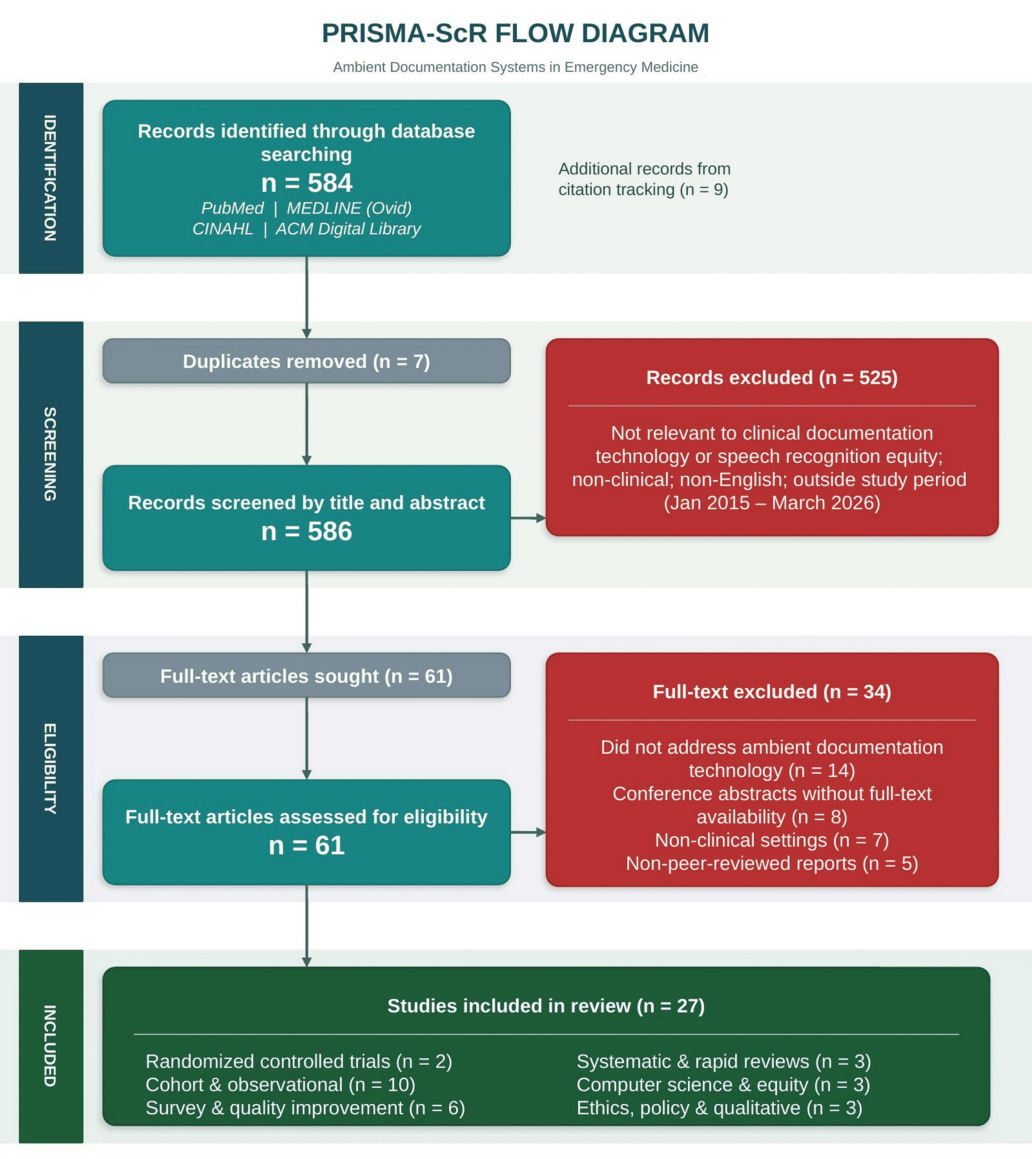
PRISMA-ScR flow diagram illustrating the selection of studies for a scoping review of ambient documentation systems in emergency medicine. Records were identified through searches of PubMed, MEDLINE (via Ovid), CINAHL, and ACM Digital Library (January 2015 - March 2026), with final searches completed on 21 March 2026. Additional records were identified through citation tracking. Database searches identified 584 records; nine additional sources were identified through citation tracking (total n = 593). After removing seven duplicates, 586 records were screened by title and abstract, of which 525 were excluded. Sixty-one full-text articles were assessed for eligibility; 34 were excluded following full-text review. A total of 27 peer-reviewed sources met all inclusion criteria and were included in the final synthesis. Included studies were categorized by design: randomized controlled trials; cohort and observational studies; survey and quality improvement studies; systematic and rapid reviews; computer science and equity analyses; and ethics, policy, and qualitative studies. This scoping review was conducted and reported in accordance with the PRISMA 2020 guidelines and PRISMA-ScR [10,11]. Abbreviations: CINAHL, Cumulative Index to Nursing and Allied Health Literature; ACM, Association for Computing Machinery; PRISMA, Preferred Reporting Items for Systematic Reviews and Meta-Analyses; PRISMA-ScR, PRISMA Extension for Scoping Reviews The figure was created using Infograpia (Infograpia, Cypress, USA), Microsoft PowerPoint (Microsoft Corp., Redmond, USA), and Adobe Acrobat (Adobe Inc., San Jose, USA).

Two reviewers (JW and SW) independently screened all titles and abstracts against the eligibility criteria in a parallel, blinded fashion. Full-text articles were retrieved for all records judged potentially eligible by either reviewer. Both reviewers then independently assessed each full-text article for final inclusion. Disagreements at either screening stage were resolved through discussion and consensus; no third adjudicator was required. The complete selection process is illustrated in the PRISMA-ScR flow diagram (Figure 1).

Data Charting Process

Data were extracted using a standardized charting form developed iteratively by the research team. Extracted fields included: author(s), year of publication, country, study design, clinical setting, sample size, intervention or technology evaluated, comparator (if applicable), key outcomes measured, and main findings. The charting form was piloted on five sources and refined before full extraction. Both reviewers contributed to data extraction; discrepancies were resolved by consensus.

Study Selection Results

The database searches identified 584 records, and an additional nine sources were identified through citation tracking, for a total of 593 records. After removing seven duplicates, 586 records underwent independent dual-reviewer screening of titles and abstracts. Of these, 525 were excluded: records did not address clinical documentation technology or speech recognition equity, focused exclusively on nonclinical settings, were not in English, or fell outside the 2015-2026 study period. Sixty-one full-text articles were assessed for eligibility. Following an independent full-text review, 34 sources were excluded: 14 for not addressing ambient documentation technology, eight for being conference abstracts without full-text availability, seven for nonclinical settings, and five for non-peer-reviewed reports. A total of 27 peer-reviewed sources met all inclusion criteria and were included in the final synthesis. The complete selection process, including specific exclusion reasons at each stage, is detailed in Figure 1. Characteristics of the included sources are summarized in Table 1.

**Table 1:** Characteristics of the included studies (n = 27). Studies are listed in order of first citation. Included sources span seven thematic areas: documentation burden and supporting evidence [5,6]; human scribes [12,13]; ambulatory ambient AI [7,14-20,32]; ED ambient AI [21-23]; ASR equity [9,24-27]; documentation precision and safety [28-30]; and financial, quality, and evidence synthesis [8,31,33]. Abbreviations: AAVE, African American Vernacular English; ASR, automatic speech recognition; AI, artificial intelligence; DAX, Dragon Ambient eXperience; ED, emergency department; EHR, electronic health record; N/A, not applicable; NASA-TLX, National Aeronautics and Space Administration Task Load Index; PDQI-9, Physician Documentation Quality Instrument; QI, quality improvement; RVU, relative value unit; VHA, Veterans Health Administration; WER, word error rate; UCI Health, University of California, Irvine Health; CORAAL, Corpus of Regional African American Language; AAL, African American Language; LOS, length of stay; KUMC, University of Kansas Medical Center; NYC, New York City; VNS Health, Visiting Nurse Service of New York; LIWC, Linguistic Inquiry and Word Count; SAE, Standard American English; HuBERT, hidden-unit bidirectional encoder representations from transformers; RoBERTa, robustly optimized bidirectional encoder representations from transformers approach; FDA, Food and Drug Administration; PCP, primary care physician; US, United States; AMA, against medical advice; SUS, System Usability Scale; AWS, Amazon Web Services; LLM, large language model; NPS, net promoter score; DR, diabetic retinopathy.

Author (Year)	Reference	Study Design	Setting	Sample Size	Technology	Key Findings
Wu et al. (2024)	[5]	Systematic review and meta-analysis	Multiple ambulatory settings	37 studies; 66,556 participants	EHR use as an exposure variable	Pooled burnout prevalence 40.4% (95% CI 37.5%-43.2%); clinicians spending >6 hours/week on EHR outside work had a significantly higher likelihood of burnout (OR 2.43, 95% CI 2.31-2.57); AI scribes identified as a proposed solution.
Arndt et al. (2024)	[6]	Longitudinal observational study	Academic primary care (EHR-integrated)	141 PCPs	Epic EHR log data (descriptive trend analysis)	Total EHR time increased (+28.4 minutes, 7.8%), inbox time increased (+14.0 minutes, 24.4%), and time on unscheduled days increased (+13.6 minutes, 19.9%).
Olson et al. (2025)	[7]	Pre-post quality improvement study	Ambulatory multispecialty; 6 health systems	263 clinicians met the final inclusion criteria	Abridge AI	Burnout decreased from 51.9% to 38.8% (OR 0.26, 95% CI 0.13-0.54); significant improvements in cognitive task load, after-hours documentation time, and focused patient attention.
Holmgren et al. (2026)	[8]	Quasi-experimental (difference-in-differences)	Ambulatory multispecialty	1,565 physicians; ~1.2M encounters	One of two commercial AI scribe tools	AI scribe adoption was associated with +1.81 RVUs/week (~$3,044 annually/physician) and +0.80 encounters/week vs. nonadopters, with no increase in claim denials.
Koenecke et al. (2020)	[9]	Corpus-based computational study	Structured sociolinguistic interview audio from CORAAL and Voices of California datasets, spanning 5 US cities	73 Black speakers, 42 White speakers; 2,141 matched audio snippets per group (19.8 hours audio)	Five commercial ASR systems (Amazon, Apple, Google, IBM, Microsoft)	All 5 ASR systems showed substantial racial disparities; average WER 0.35 for Black speakers vs. 0.19 for White speakers; >20% of Black speaker snippets produced unusable transcripts vs. 1.6% for White speakers; disparities traced to acoustic models, not language models; AAVE dialect density positively correlated with error rate.
Hess et al. (2015)	[12]	Prospective quasi-experimental pre-post design	2 EDs	Approximately 70 providers	Human scribes	36% reduction in time spent documenting (44% to 28% of shift); 30% increase in time spent in direct patient contact (37% to 48%); RVUs/hour increased 5.5% and RVUs/patient increased 5.3% (both statistically significant); rates of patients leaving AMA decreased; LOS unchanged despite 5% volume increase.
Ullman et al. (2021)	[13]	Systematic review	Emergency medicine	20 studies	Human scribes	Scribes may increase patients seen per shift and decrease LOS (low certainty; estimated ~1 additional patient per 10-hour shift); results were mixed for door-to-room/provider times, patients left without being seen, and patient/clinician satisfaction; no data on documentation quality or medical errors; implementation costs (hiring, training, supervision) are substantial and chronically underreported in the literature; all included studies used in-person rather than virtual scribes.
Pearlman et al. (2025)	[14]	Pre-post cohort	Multispecialty ambulatory	125 users; 478 controls	Abridge ambient AI scribe (3-month pilot)	8.5% reduction in mean EHR time/appointment (2.4 min; 95% CI -12.8% to -3.9%); 15.9% reduction in mean note time/appointment (1.8 min; 95% CI -21.2% to -10.4%); no significant changes in after-hours documentation, appointment length, or volume in propensity score analysis; dose-response relationship observed; greatest gains among female clinicians, primary care, and medical subspecialists.
Albrecht et al. (2025)	[15]	Pre-post quality improvement survey	Single academic medical center (KUMC), ambulatory multispecialty (30 specialties)	181 enrolled; 99 post-survey respondents	Abridge AI	6.91x higher odds of finding documentation workflow easy post-implementation (OR 6.91, 95% CI 3.90-12.56); 4.95x higher odds of completing notes before next patient visit (OR 4.95, 95% CI 2.87-8.69); 73% agreed after-hours documentation decreased; 67% agreed burnout risk reduced; 64% agreed job satisfaction increased; effects did not differ by specialty or duration of use.
Hudson et al. (2025)	[16]	Pre-post quality improvement	Academic medical center, ambulatory multispecialty	40 providers	Abridge AI	Use of Abridge was associated with significantly lower NASA-TLX composite scores and reductions across all three subdimensions - effort, mental demand, and temporal demand - all p < 0.001, indicating a substantial reduction in cognitive load.
Shah et al. (2025)	[17]	Prospective single-group pre-post quality improvement study	Ambulatory multispecialty	48 physicians	DAX Copilot	Significant reductions in physician task load (-24.42, p < 0.001) and burnout/work exhaustion (-1.94, p < 0.001); improved system usability score (+10.9, p = 0.003); median perceived time savings 20 min/half-day.
Duggan et al. (2025)	[18]	Prospective single-group pre-post quality improvement study with EHR metrics and surveys	Multispecialty ambulatory	46 clinicians	DAX Copilot (Nuance)	20.4% less time in notes (p < 0.001); 9.3% more same-day closure (p < 0.001); 30.0% less after-hours work (p = 0.02); mixed satisfaction (NPS = 0); note length increased 20.6%.
Lukac et al. (2025)	[19]	Parallel three-group pragmatic randomized clinical trial	Multispecialty ambulatory	238 physicians (79 DAX, 79 Nabla, 80 control)	Microsoft DAX Copilot v2.0 and Nabla v1.5	Nabla: 9.5% reduction in time-in-note vs. control (p = 0.02); DAX: no significant difference (p = 0.66); no significant change in after-hours EHR time for either tool; secondary psychometric outcomes (burnout, task load) trended positive but require confirmation.
Afshar et al. (2025)	[20]	24-week stepped-wedge individually randomized pragmatic clinical trial	Multispecialty ambulatory	66 practitioners; 71,000+ notes	Abridge AI	Significant reduction in work exhaustion/interpersonal disengagement (-0.44 points, p < 0.001); nonsignificant increase in professional fulfillment (+0.14, p = 0.04); -0.36 hours/day documentation time; ICD-10 billing compliance improved (p < 0.001).
Morey et al. (2025)	[21]	Prospective pilot	Single ED	710 encounters (284 human scribe, 426 AI scribe)	Abridge AI	AI scribes associated with more time in the notes section (adult: 4.3 vs. 1.8 min, p < 0.001); note quality was similar for adults but lower for pediatric patients (p = 0.02); physicians contributed significantly more note characters with AI; no physician satisfaction measure was included.
Preiksaitis et al. (2026)	[22]	Retrospective observational study	Academic ED	8,740 eligible encounters; 976 (11.2%) ambient, 7,764 standard	DAX Copilot	11.2% overall adoption rate; highly skewed - top 10% of users accounted for 70.5% of ambient encounters; ambient AI associated with 28% reduction in on-shift documentation time (2:45 vs. 3:50 min); 16% reduction in total EHR time; shorter notes; adoption clustered in lower-acuity, non-interpreted encounters.
Webb et al. (2026)	[23]	Prospective crossover trial	Academic ED	20 enrolled, 18 completed both phases	DAX Copilot and Abridge AI - both tools evaluated head-to-head	DAX was associated with greater perceived burden reduction (Likert median 1.5 vs. 2, p = 0.025); usability was high and comparable (SUS medians both 73.5, p = 0.94); DAX scored higher on modified PDQI-9 (39 vs. 36.5, p = 0.011); satisfaction differences favoring DAX disappeared after adjusting for order effects; no overall tool preference after controlling for order.
Zolnoori et al. (2024)	[24]	Computational/observational accuracy study	Home healthcare patient-nurse encounters, NYC (VNS Health)	35 patients (16 Black patients, 19 White patients); 860 utterances from 10-patient subsample	AWS General Transcribe, AWS Medical Transcribe, Whisper, Wave2Vec	Best overall accuracy: AWS General (median WER 39%); all systems performed worse for Black patients (AWS General: WER 50% Black patients vs. 33% White patients, p = 0.016); greatest LIWC discrepancies in Affect, Social, and Drives dimensions for Black patients; Wave2Vec excluded from further analysis due to near-total failure (median WER 91%).
Martin and Wright (2023)	[25]	Theoretical review integrating sociolinguistic and computational literature	Literature review - no primary data collection	N/A	Multiple ASR systems reviewed (Amazon, Apple, Google, IBM, Microsoft, DeepSpeech)	Documented racial bias against AAL speakers in ASR systems; synthesized phonological and morphosyntactic AAL features driving ASR failure (invariant 'be', metathesis, prosody); described allocational and representational harms in employment and healthcare; called for cross-disciplinary collaboration to address bias.
Harris et al. (2024)	[26]	Computational empirical study	Nonclinical podcast audio (Spotify dataset); four US English dialect groups (SAE, AAVE, Chicano English, Spanglish)	92 speakers; 13 hours of annotated audio	Whisper (multiple sizes), HuBERT, Wav2vec 2.0 - evaluated zero-shot and fine-tuned	SAE consistently outperformed all minority dialects across every model; within minority dialect groups, women outperformed men, while men SAE speakers outperformed women - suggesting men of color using minority dialects face the highest ASR error risk; fine-tuning improved but did not eliminate disparities.
Ezema et al. (2025)	[27]	Computational study with real-world educational data	High-dosage tutoring program (educational, not clinical); Black and White tutors in Title I schools	34 tutors; 88 sessions; 12,572 utterances	Whisper large-v2 ASR (open-source); RoBERTa discourse classifier	Whisper ASR had ~24% higher error rate for Black speakers vs. White speakers (acoustic, not linguistic). ASR errors cascaded to downstream AI feedback, masking superior discourse quality in Black speakers. Fine-tuning reduced but did not eliminate the gap.
Brunner et al. (2026)	[28]	Mixed-methods study (quantitative PDQI-9 ratings + qualitative focus groups)	4 specialties, VHA	16 specialist clinicians; 82 PDQI-9 survey responses	Two LLM-based ambient scribing solutions (A and B), tested in standardized simulated patient encounters	Mean note quality 36.2/50; highest in neurology (41.2) and lowest in cardiology (32.0), not significant (p = 0.09). Hallucinations, overconfident diagnoses, and distorted physical exam findings were cross-specialty concerns.
Topaz et al. (2025)	[29]	Expert commentary	Clinical practice	N/A	AI scribes broadly	Identified patient safety risks including AI hallucinations, critical omissions, misattribution, and contextual misinterpretations; documented racial/linguistic ASR disparities disproportionately affecting Black patients; raised concerns about algorithmic opacity, absence of FDA oversight, and unconsented secondary use of patient data; called for rigorous validation, mandatory transparency, updated liability frameworks, and equity-focused implementation.
Gerke et al. (2020)	[30]	Ethics/legal analysis	Healthcare AI	N/A	Healthcare AI broadly - including diagnostic imaging systems (IDx-DR, OsteoDetect), clinical decision support (IBM Watson for Oncology), and AI health apps (Ada, Corti); not specific to ambient documentation scribes	Mapped four primary ethical challenges (informed consent, safety and transparency, algorithmic fairness and bias, data privacy) and five legal challenges (safety and effectiveness, liability, data protection and privacy, cybersecurity, intellectual property law) across US and European regulatory frameworks; emphasized the need for stakeholder collaboration to ensure ethical and legally compliant AI implementation.
Kanaparthy et al. (2025)	[31]	Rapid review	Multiple healthcare settings	6 studies meeting criteria from 1,450 identified	Digital scribes broadly (ambient listening + generative AI)	Consistent reductions in self-reported documentation time; notes increased in length; physician burnout (standardized scales) unaffected but engagement improved; productivity (billing metrics) unchanged; real-world multifaceted evidence needed before unequivocal clinical recommendation.
Guo et al. (2026)	[32]	Mixed-methods QI study	Multispecialty ambulatory (UCI Health)	167 physicians (EHR metrics); 65 physicians (survey)	DAX Copilot and Abridge AI pooled data	~14% reduction in daily documentation time; reduced cognitive demand (p = 0.031) and documentation effort (p = 0.014); improved patient-centered care perceptions; note length modestly increased; no significant change in same-day encounter closure rates; specialty-specific customization identified as key area for improvement; vendor-specific differences could not be assessed within the QI framework.
Van Tiem et al. (2026)	[33]	Qualitative/ethnographic study	Academic medical center (University of Iowa)	24 clinicians	Nabla ambient scribe	Clinicians felt more present with patients and reported greater satisfaction; AI-drafted notes introduced a perceived loss of clinical "voice" and unfamiliar formatting; inpatient and procedure-heavy settings showed limited benefit where documentation was already highly standardized; the genre theory framework was applied to interpret sociotechnical dimensions of documentation work.

Results

Synthesis of Results

Included sources comprised two randomized controlled trials (RCTs), 10 cohort and observational studies, six survey and quality improvement studies, three systematic or rapid reviews, three computer science and equity analyses, and three ethics, policy, and qualitative studies. Results were synthesized narratively and organized thematically, consistent with PRISMA-ScR guidance. Thematic areas identified a priori and refined during extraction were: (1) human scribes as baseline comparators; (2) physician workflow, cognitive load, and burnout, presented across ambulatory and multicenter or randomized trial evidence; (3) documentation accuracy and precision; (4) patient experience, consent, and trust; (5) ED throughput and scalability; and (6) algorithmic equity and ASR bias. Formal critical appraisal of individual sources was not performed, consistent with scoping review methodology, and no meta-analysis was conducted; the synthesis was descriptive. Where quantitative outcomes were cited, key statistical measures reported in the original studies were included.

Human Scribes as Baseline Comparators

Human scribes have been adopted in EDs to mitigate documentation burden, establishing a performance baseline against which ambient systems are compared. Hess et al. evaluated a scribe program at two academic EDs with a combined volume of 100,000 annual patient visits and found a 36% reduction in time spent documenting and a 30% increase in time spent in direct patient contact, with relative value units (RVUs) per hour and per patient both increasing significantly [12]. A systematic review by Ullman et al. of 20 studies concluded that in-person medical scribes may improve ED efficiency and financial productivity, though the certainty of evidence was low, effect sizes on efficiency were small, and results for door-to-room time, patients leaving without being seen, and patient and clinician satisfaction were mixed [13]. Notably, no studies in the Ullman review examined documentation quality or medical errors, and the costs of developing, implementing, and maintaining scribe programs were rarely reported. Human scribe programs face significant scalability limits: recruitment pipelines, one-to-one staffing ratios, geographic constraints, and staff turnover make system-wide deployment impractical and motivate the evaluation of ambient alternatives.

Ambulatory Evidence: Physician Workflow, Cognitive Load, and Burnout

The strongest evidence addresses the impact of ambient AI on physician workflow efficiency and clinician well-being, primarily from ambulatory settings. Pearlman et al. studied 125 AI scribe users and 478 controls and found an 8.5% reduction in mean EHR time per appointment and a 15.9% reduction in note time per appointment, with benefits most pronounced among female clinicians, primary care providers, and medical subspecialists [14]. Albrecht et al. reported significant improvements in documentation workflow ease (OR 6.91; 95% CI 3.90-12.56) and timely note completion (OR 4.95; 95% CI 2.87-8.69) across 181 clinicians at a multispecialty academic medical center using Abridge [15]. Hudson et al. studied 40 ambulatory providers and found a 46.6% reduction in National Aeronautics and Space Administration Task Load Index (NASA-TLX) composite score (221.20 vs. 118.20), with significant reductions across all three subdimensions, including effort (46.3%), mental demand (48.8%), and temporal demand (44.4%), all p < 0.001, indicating a substantial reduction in cognitive load [16]. Shah et al. similarly reported a 24.42-point reduction in physician task load (p < 0.001), a 1.94-point reduction in burnout/work exhaustion (p < 0.001), and an improved system usability score (+10.9, p = 0.003) in a DAX Copilot pilot involving 48 physicians, with a median perceived time savings of 20 minutes per half-day of clinic [17]. Duggan et al. found that ambient scribe use was associated with greater documentation efficiency, including 20.4% less time in notes, 30.0% less after-hours work, and 9.3% more same-day note closure, though overall clinician satisfaction was mixed (net promoter score = 0) [18].

Multicenter and Randomized Trial Evidence

The largest evidence base to date comes from a multicenter study by Olson et al. of 263 clinicians across six health systems: self-reported burnout fell from 51.9% to 38.8% (OR 0.26; 95% CI 0.13-0.54), with significant improvements in cognitive task load, after-hours documentation, and focused patient attention [7]. Two RCTs, however, now provide the highest level of evidence. Lukac et al. conducted a parallel three-group pragmatic RCT randomizing 238 physicians to Microsoft DAX Copilot, Nabla, or usual care; Nabla users achieved a 9.5% reduction in time-in-note versus control (p = 0.02), while DAX showed no significant difference in this outcome (p = 0.66); secondary psychometric outcomes including burnout and task load trended positive for both platforms but require confirmation [19]. Afshar et al. conducted a 24-week stepped-wedge pragmatic RCT with 66 practitioners and over 71,000 notes at an academic health system spanning ambulatory clinics in Wisconsin and Illinois, demonstrating significant reductions in work exhaustion and interpersonal disengagement (-0.44 points; p < 0.001), a 0.36-hour daily reduction in documentation time, and improved International Classification of Diseases, Tenth Revision (ICD-10) billing compliance [20]. Together, these trials confirm that ambient AI scribes reduce documentation burden and support clinician well-being, though the magnitude and consistency of effect vary across platforms and outcome measures.

Documentation Accuracy and Precision

Documentation precision, encompassing accuracy, completeness, internal consistency, and auditability, is a central concern for clinical deployment of ambient systems. Evidence on note quality is mixed but informative. Morey et al. compared ambient AI (Abridge) with human scribes across 710 ED encounters and found comparable note quality scores for adult patients; however, note quality was lower for pediatric encounters, and physicians contributed significantly more note characters with AI assistance than with human scribes, spending more time in the notes section of the EHR [21]. Preiksaitis et al. found that ambient AI was associated with shorter note length in ED encounters, which may reflect improved efficiency or could indicate omission of clinically relevant content [22]. Webb et al., in a crossover trial comparing DAX and Abridge among 18 emergency physicians, found that DAX scored significantly higher on the modified Physician Documentation Quality Instrument (PDQI-9) (39 vs. 36.5; p = 0.011), though both tools demonstrated high and comparable usability scores [23]. Beyond clinician-level documentation variation, transcription inaccuracy introduced by ASR systems may compromise note fidelity, particularly for underrepresented speaker groups, an equity dimension explored in detail in the Algorithmic Equity section below [24-27].

Brunner et al. evaluated ambient scribe technology across four specialties (cardiology, gastroenterology, hematology-oncology, and neurology) at a Veterans Health Administration simulation center, using PDQI-9 scoring combined with qualitative focus groups across standardized simulated patient encounters [28]. The study identified a nonsignificant trend toward inter-specialty variability in documentation quality, with neurology rating highest (41.2/50) and cardiology lowest (32.0/50) (p = 0.09). Cardiology's underperformance was attributed primarily to the absence of EHR integration with prior imaging and procedural data, rather than procedure volume per se. The controlled simulation setting provided an assessment independent of real-world adoption variation.

Several risks are documented at the trial level. In the Lukac et al. RCT, secondary psychometric outcomes trended favorably but did not reach the threshold for confirmation, and the authors emphasized that active physician oversight, not passive acceptance of AI-generated text, is essential [19]. Topaz et al. identified further documentation risks, including content misattribution, selective omissions, and the accountability challenge of assigning clinical and legal responsibility when AI drafts notes [29]. Gerke et al. similarly highlight the importance of transparency, privacy protections, and defined clinician responsibility in AI-assisted documentation [30]. A recent rapid review concluded that while digital scribes show promise, well-designed real-world studies are needed before AI scribes can be recommended unequivocally [31]. Guo et al. similarly reported modest note length increases alongside documentation time reductions in a multispecialty quality improvement study; these findings are discussed in detail in the ED Throughput section below and raise comparable questions about whether efficiency gains come at the cost of note completeness [32].

Beyond efficiency metrics, qualitative evidence suggests that ambient AI may shift rather than resolve documentation quality concerns. Van Tiem et al. conducted an ethnographic study of 24 clinicians across a pilot and enterprise-wide rollout of the Nabla ambient scribe, using semi-structured interviews analyzed through a documentation genre framework [33]. Clinicians described meaningful benefits in patient engagement and perceived efficiency, but also expressed significant ambivalence: ambient-generated notes were frequently characterized as too verbose in the History of Present Illness, underspecified in the Assessment and Plan, and inconsistent with individual documentation voice. Participants described how note-writing does more than document encounters; it signals expertise, manages clinical impressions, conveys calibrated assessments to referring clinicians, and teaches trainees how to reason clinically. Inpatient and procedure-heavy settings reported limited benefit where documentation was already highly templated. These findings suggest that evaluation frameworks relying solely on efficiency metrics may miss clinically meaningful dimensions of documentation work.

Patient Experience, Consent, and Trust

Ambient documentation systems show early promise for improving the patient-facing dimension of clinical encounters. In the Olson et al. multicenter study, clinicians reported significant improvement in their ability to provide focused patient attention after the implementation of ambient AI [7]. Albrecht et al. further found that among post-survey respondents, 77% agreed the tool improved patient care by decreasing documentation burden, 73% agreed that after-hours documentation decreased, 67% agreed that burnout risk was reduced, and 64% agreed that job satisfaction increased [15]. Duggan et al. found that ambient scribing was associated with significantly lower sense of distraction during patient conversations and reduced cognitive burden from documentation among 46 clinicians, suggesting that time freed from EHR tasks supported more engaged patient interaction [18].

Recording clinical conversations introduces important ethical and practical questions about privacy, consent, and trust. Patients may receive only minimal disclosure that recording is occurring, without meaningful details about speech processing, audio storage, or third-party vendor access [29]. EDs face particular challenges: patients may be intubated, cognitively altered, or in extremis and unable to provide meaningful consent. Acuity-appropriate consent workflows for ambient documentation in emergency medicine remain an unresolved implementation challenge that future research and institutional policy must address.

ED Throughput, Scalability, and Financial Implications

Three studies examined ambient AI specifically in ED settings. Morey et al. provide the earliest comparative data, finding comparable note quality for adult patients when comparing Abridge against human scribes across 710 encounters; however, physicians using ambient documentation contributed significantly more note characters and spent more time actively in the notes section than their counterparts using human scribes [21]. These findings suggest that ambient documentation may shift rather than eliminate documentation effort, particularly in emergency settings characterized by interruptions, multitasking, and evolving clinical narratives.

Preiksaitis et al. studied 8,740 adult ED encounters at an academic tertiary center and found that overall ambient AI adoption was 11.2%, but highly variable across physicians, the top 10% of users accounting for 70.5% of ambient encounters. Adoption was clustered in lower-acuity, non-interpreted encounters [22]. When engaged, ambient AI was associated with a 28% reduction in on-shift documentation time (2:45 vs. 3:50 minutes) and a 16% reduction in total EHR time, with shorter note length. This preference for lower-acuity encounters likely reflects legitimate concerns about transcription reliability in high-noise, multi-provider environments and the added complexity of interpreter-mediated, multilingual encounters. Webb et al. conducted a prospective crossover trial comparing DAX and Abridge among 18 emergency physicians who completed both phases; DAX was associated with greater perceived work burden reduction on a Likert scale (median 1.5 vs. 2.0; p = 0.025) [23].

Guo et al. conducted a mixed-methods quality improvement study across 167 physicians at the University of California, Irvine Health (UCI Health), combining EHR time-efficiency metrics with pre- and post-implementation surveys [32]. Documentation time decreased approximately 14% overall, while note length modestly increased. Survey results confirmed reduced cognitive demand (p = 0.031) and documentation effort (p = 0.014), with specialty-specific customization identified as a key area for refinement, reinforcing that efficiency gains are distributed unevenly across clinical contexts.

Beyond individual encounter efficiency, ambient systems offer a structural scalability advantage over human scribe programs. The Olson et al. study demonstrated simultaneous deployment across six health systems [7], and University of Wisconsin Health (UW Health) subsequently expanded the ambient AI system-wide following the Afshar et al. trial [20], a scale that would require years of hiring and training to replicate with human scribe programs [12,13]. Financial data reinforce these benefits: Holmgren et al. analyzed nearly 1.2 million ambulatory encounters across 1,565 physicians and found that ambient AI adopters generated 1.81 additional RVUs per week, translating into approximately $3,044 in additional annual revenue per physician, with no increase in claim denials [8]. Afshar et al. also documented improved ICD-10 billing compliance alongside well-being benefits, suggesting concurrent quality and financial gains [20].

Algorithmic Equity and ASR Bias

Ambient documentation systems depend on ASR as their foundational technology, and the computer science literature documents substantial performance disparities with direct clinical relevance. Koenecke et al. evaluated five major commercial ASR systems (Amazon, Apple, Google, IBM, Microsoft) and found that all exhibited significant racial disparities: the average WER was 0.35 for Black speakers versus 0.19 for White speakers, with more than 20% of Black speaker audio snippets producing unusable transcripts compared with only 1.6% for White speakers [9]. These disparities were traced to acoustic models trained on unrepresentative datasets, not to language models, and African American Vernacular English (AAVE) dialect density was positively correlated with error rate.

These findings have been confirmed and extended in healthcare-specific contexts. Zolnoori et al. evaluated four ASR systems, including Amazon Web Services (AWS) General Transcribe, AWS Medical Transcribe, Whisper, and Wave2Vec, for patient-nurse communication in home healthcare and found lower transcription accuracy for Black patients across all systems, with the best-performing system (AWS General Transcribe) still yielding a median WER of 50% for Black patients versus 33% for White patients (p = 0.016) [24]. Martin and Wright demonstrated that ASR bias is embedded in the architectural design of these systems through specific phonological and morphosyntactic features of African American Language that current acoustic models systematically fail to capture [25]. Harris et al. found compounding bias at the intersection of gender and dialect, with speakers of African American English, Chicano English, and Spanglish experiencing significantly higher error rates, and with men of color using minority dialects facing the greatest ASR error risk [26]. Ezema et al. confirmed algorithmic bias in ASR across multiple demographic groups in real-world educational data, calling for systematic bias auditing prior to clinical deployment [27].

EDs serve some of the most linguistically and racially diverse patient populations in healthcare, which gives these disparities direct clinical implications. Errors in ambient transcription of history of present illness narratives, medication names, and dosages could disproportionately affect documentation quality for Black, Hispanic, and non-native English-speaking patients, potentially contributing to documentation-level health disparities. Unlike human biases, however, algorithmic biases can be systematically identified, monitored, and improved. Koenecke et al. proposed that training on more linguistically diverse datasets could reduce disparities [9], and ongoing equity auditing should be a core component of ambient AI governance frameworks.

Proposed Seven-Domain Framework

**Figure 2:**
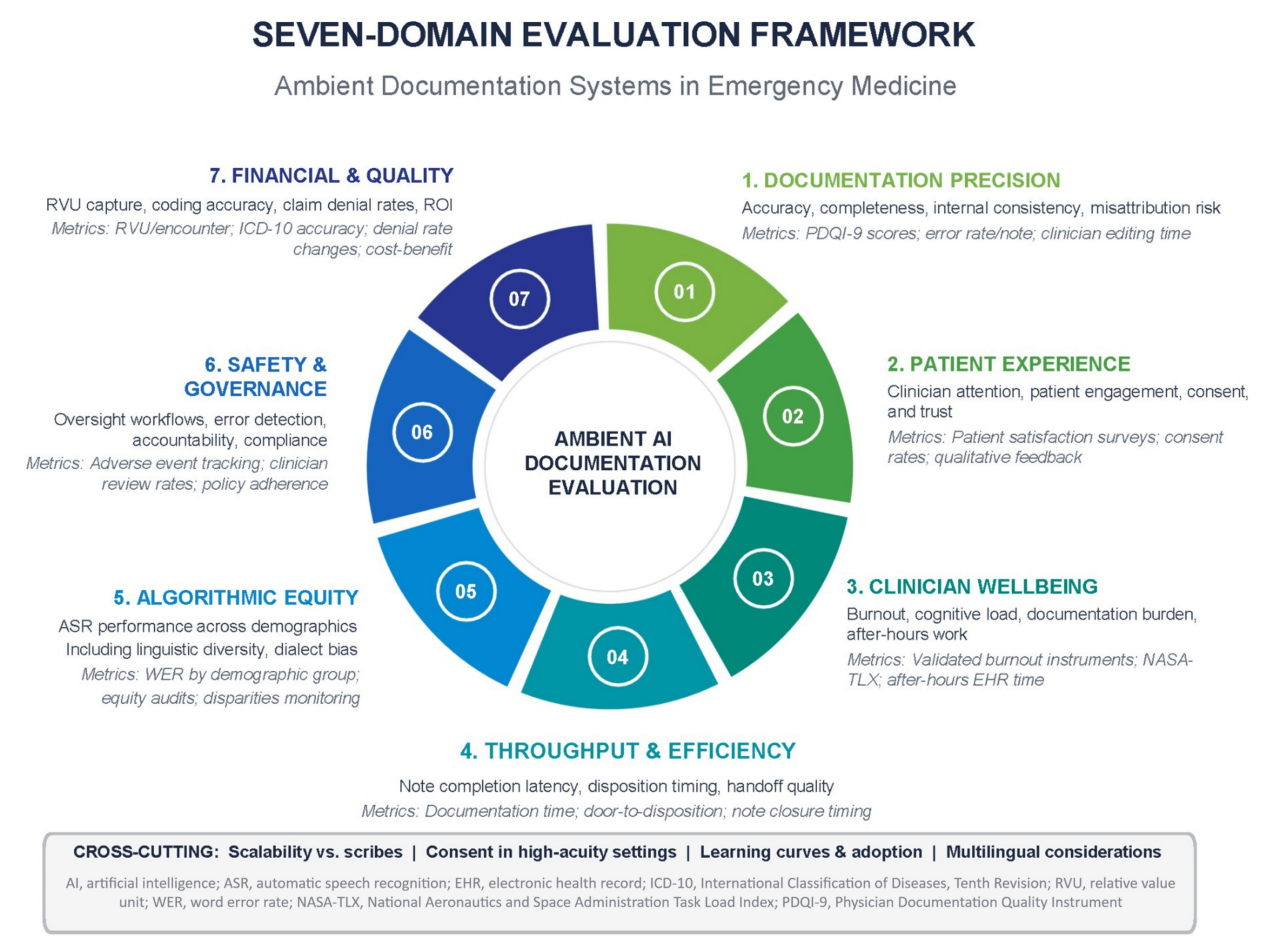
Seven-domain evaluation framework for ambient documentation systems in emergency medicine. Domains encompass documentation precision, patient experience, clinician well-being, throughput and efficiency, algorithmic equity, safety and governance, and financial and quality outcomes. Each domain includes representative metrics for evaluating ambient AI scribe performance in emergency department settings. Cross-cutting considerations include scalability relative to human scribes, consent workflows in high-acuity settings, learning curves and adoption patterns, and multilingual documentation challenges. References: Framework domains synthesized from included studies [5-9,12-33]. Domain 1 informed by Topaz et al. [29] and Brunner et al. [28]. Domain 3 informed by Olson et al. [7], Albrecht et al. [15], and Guo et al. [32]. Domain 5 informed by Koenecke et al. [9], Zolnoori et al. [24], Martin and Wright [25], Harris et al. [26], and Ezema et al. [27]. Domain 7 informed by Holmgren et al. [8]. The figure was created using Infograpia (Infograpia, Cypress, USA), Microsoft PowerPoint (Microsoft Corp., Redmond, USA), and Adobe Acrobat (Adobe Inc., San Jose, USA).

Based on the thematic areas identified across this review, we propose a seven-domain evaluation framework for ambient documentation systems in emergency medicine (Figure 2). The seven domains are: (1) documentation precision, encompassing accuracy, completeness, and misattribution risk; (2) patient experience, including clinician attention, consent processes, and patient trust; (3) clinician well-being, covering burnout, cognitive load, and after-hours documentation burden; (4) throughput and efficiency, addressing note completion latency, disposition timing, and handoff quality; (5) algorithmic equity, examining ASR performance across racial, ethnic, and linguistic groups; (6) safety and governance, comprising oversight workflows, error detection, accountability, and regulatory compliance; and (7) financial and quality outcomes, including RVU capture, coding accuracy, and return on investment. The framework expands from six thematic areas to seven domains by elevating safety and governance and financial and quality outcomes as distinct evaluative dimensions, both of which emerged consistently across the included literature as operationally critical yet underreported in prior evaluations. Cross-cutting considerations that span all domains include scalability relative to human scribes, consent workflows in high-acuity settings, learning curves and adoption patterns, and multilingual documentation challenges. This framework is conceptual, derived from narrative synthesis consistent with scoping review methodology, and intended to guide future research and implementation rather than serve as a validated measurement instrument. Prior evaluations have applied general healthcare AI frameworks such as Quality of Information, Understanding and Reasoning, Expression Style and Persona, Safety and Harm, Trust and Confidence (QUEST) and Systems Engineering Initiative for Patient Safety v3.0 (SEIPS 3.0) to digital scribes [31]; the present framework extends those approaches by centering ED-specific operational conditions, including high-acuity interruption-dense workflows, interpreter-mediated encounters, and the unique consent challenges of cognitively impaired or critically ill patients.

## Conclusions

Ambient documentation systems offer a scalable, promising approach to addressing documentation burden in emergency medicine. The ambulatory evidence base demonstrates largely consistent benefits in reducing EHR time, cognitive load, and clinician burnout, though effect sizes and statistical significance vary across platforms and study designs, and at least one randomized trial found no significant reduction in documentation time for one of the two platforms evaluated. ED-specific studies support the feasibility of ambient AI, with adoption currently concentrated in lower-acuity, non-interpreted encounters; the performance and uptake of ambient documentation in high-acuity, complex ED settings represent an important area for future investigation. The scalability of ambient systems over human scribe programs represents a practical advantage for health systems seeking relief from organization-wide documentation burden, particularly in EDs, where staffing constraints make consistent human scribe coverage logistically challenging. Equitable deployment requires attention to disparities in ASR across racial, ethnic, and linguistic groups, with ongoing monitoring and bias auditing as core components of governance. Future research should focus on ED-specific outcomes, including documentation accuracy and patient safety, long-term safety monitoring, patient-centered consent processes, financial and quality implications, and strategies to ensure that ambient documentation benefits all patient and clinician populations equitably. Systematic progress across the seven proposed domains, from documentation precision and clinician well-being through algorithmic equity, safety, and governance, and financial and quality outcomes, will be essential to realizing the full potential of ambient documentation in emergency medicine responsibly and inclusively.

## Appendices

Appendix A: Full database search strategies

The following search strategies were used for each database. All searches were conducted between January 2015 and March 2026, with final searches completed on 21 March 2026.

PubMed/MEDLINE (Ovid)

Search terms: ("ambient artificial intelligence"[tiab] OR "ambient documentation"[tiab] OR "digital scribe"[tiab] OR "AI scribe"[tiab] OR "ambient scribe"[tiab] OR "automatic speech recognition"[MeSH Terms] OR "clinical documentation"[tiab]) AND ("emergency department"[tiab] OR "emergency medicine"[MeSH Terms] OR "physician burnout"[tiab] OR "burnout, professional"[MeSH Terms] OR "electronic health records"[MeSH Terms] OR "documentation burden"[tiab] OR "speech recognition bias"[tiab] OR "speech recognition disparities"[tiab]). Filters: English language; publication date 2015/01/01-2026/03/21; peer-reviewed journals.

Cumulative Index to Nursing and Allied Health Literature (CINAHL)

Search terms: (TX "ambient AI" OR TX "ambient documentation" OR TX "digital scribe" OR TX "AI scribe" OR TX "ambient scribe" OR TX "automatic speech recognition") AND (TX "emergency department" OR TX "physician burnout" OR TX "EHR burden" OR TX "documentation burden" OR TX "speech recognition disparities"). Limiters: peer reviewed; English language; published date: 20150101-20260321.

Association for Computing Machinery (ACM) Digital Library

Search terms: (acmdlTitle:("ambient documentation" OR "ambient AI scribe" OR "digital scribe" OR "AI scribe" OR "automatic speech recognition") OR Abstract:("ambient AI" OR "ambient documentation" OR "digital scribe")) AND (Abstract:("emergency department" OR "emergency medicine" OR "physician burnout" OR "documentation burden" OR "speech recognition bias" OR "speech recognition disparities" OR "ASR disparities")). Filters: published 2015-2026; English language. Results screened by title and abstract for relevance; citation tracking performed for key identified articles.
